# Supplementary material for: Green Walkability and Physical Activity in UK Biobank: A Cross-Sectional Analysis of Adults in Greater London
Source: Int J Environ Res Public Health. 2022 Apr 2;19(7):4247. doi: 10.3390/ijerph19074247 (PMC8998308; doi:10.3390/ijerph19074247)
Supplement: Supplementary file 1 [file ijerph-19-04247-s001.zip › ijerph-1639476-supplementary.pdf]

Supplementary Material – Green walkability and physical activity in UK Biobank

Contents

Table S1: Ordnance Survey Points of Interest (POI) categories used in *walkability* and *green walkability* index to represent density of potential destinations surround each UK Biobank participant residential address.
Table S2-S17: Table 1 by quintiles of walkability, green walkability, ground-cover walkability and tree-cover walkability for UK Biobank participants in the samples for the IPAQ based self-reported analyses (n = 57,726), accelerometer measured physical activity (n = 12,986), non-commute transport (n = 45,181) and commuting transport analyses (n = 24,102).

Table S1: Ordnance Survey Points of Interest (POI) categories used in *walkability* and *green walkability* index to represent density of potential destinations around each UK Biobank participant residential address.

Group	Category	Class
**01 Accomodations, eating and drinking**	02 Eating and drinking	0012 Banqueting and function rooms
		0013 Cafes, snack bars and tea rooms
		0018 Fast food and takeaway outlets
		0019 Fast food delivery services
		0020 Fish and chip shops
		0025 Internet cafes
		0034 Pubs, bars and inns
		0043 Restaurants
**02 Commercial Services**	08 IT, advertising, marketing and media services	0130 Printing and photocopying services
	09 Legal and financial	0138 Banks and building societies
		0141 Cash machines
		0140 Currency conversion and money transfers
		0151 Pawnbrokers
		0154 Solicitors, advocates and notaries public
	10 Personal, consumer and other services	0112 Event ticket agents and box office
		0156 Hair and beauty services
		0822 Slimming clubs and services
		0821 Spas
		0179 Sports services
		0776 Tailoring and clothing alteration
**03 Attractions**	58 Bodies of water	0804 Lakes and waters
		0805 Lochs and lochans
		0803 Ponds
		0807 Reservoirs
		0808 Settling, balancing and silt ponds
		0806 Tarns, pools and meres
	16 Botanical and zoological	0231 Aquaria and sea life centres
		0232 Bird reserves, collections and sanctuaries
		0233 Butterfly farms
		0235 Farm based attractions
		0236 Horticultural attractions
		0237 Salmon ladders
		0239 Zoos and animal collections
	17 Historical and cultural	0240 Archaeological sites
		0813 Art galleries
		0241 Battlefields
		0245 Historic and ceremonial structures
		0244 Historic buildings including castles, forts and
		abbeys
		0246 Historical ships
		0248 Museums
	19 Landscape features	0257 Designated scenic features 0259 Trigonometric points
	18 Recreational	0252 Commons
		0253 Country and National Parks

		0814 Municipal parks and gardens
		0254 Picnic areas
		0255 Playgrounds
	20 Tourism	0268 Information centres
		0263 Laseria, observatories and planetaria
		0264 Model villages
		0265 Railways (heritage, steam and miniature)
		0267 Siteseeing, tours, viewing and visitor centres
		0266 Theme and adventure parks
		0269 Unspecified and other attractions
**04 Sport and entertainment**	22 Gambling	0277 Amusement parks and arcades
		0278 Bingo halls
		0279 Bookmakers
		0280 Casinos
		0281 Pools promoters
	23 Outdoor pursuits	0282 Angling and sports fishing
		0283 Combat, laser and paintball games
		0284 Hot air ballooning
		0770 Outdoor pursuit organisers and equipment
		0285 Parachuting and bungee jumping
		0286 Paragliding and hang gliding
		0321 Riding schools, livery stables and equestrian centres
		0287 Watersports
	21 Sport and entertainment support services	0271 Children's activity centres
		0273 Entertainment services
		0274 Firework related services
		0275 Funfair services
		0276 Mobile discos
		0820 Motorsport services
	24 Sports complex	0289 Athletics facilities
		0290 Bowling facilities
		0291 Climbing facilities
		0292 Golf ranges, courses, clubs and professionals
		0293 Gymnasiums, sports halls and leisure centres
		0294 Ice rinks
		0297 Motorsport venues
		0298 Racecourses and greyhound tracks
		0299 Shooting facilities
		0300 Ski infrastructure and aerial cableways
		0301 Snooker and pool halls
		0302 Sports grounds, stadia and pitches
		0303 Squash courts
		0304 Swimming pools
		0305 Tennis facilities
		0306 Velodromes
	25 Venues, stage and screen	0825 Adult venues
		0308 Cinemas
		0762 Conference and exhibition centres
		0311 Discos
		0312 Nightclubs
		0314 Social clubs
		0315 Theatres and concert halls
**05 Education and health**	26 Animal welfare	0316 Animal clipping and grooming
		0317 Dog training
		0318 Horse training
		0319 Kennels and catteries

		0320 Pet cemeteries and crematoria
		0322 Veterinarians and animal hospitals
		0323 Veterinary pharmacies
	27 Education support services	0324 Education authorities
		0325 Education services
		0326 Examination boards
		0799 Playing for success centres
		0800 Secure units
	28 Health practitioners and establishments	0780 Accident and emergency hospitals
		0330 Alternative, natural and complementary
		0364 Chemists and pharmacies
		0365 Clinics and health centres
		0815 Day and care centres
		0367 Dental and medical laboratories
		0368 Dental surgeries
		0335 Dental technicians
		0337 Dieticians and nutritionists
		0369 Doctors surgeries
		0333 Foot related services
		0340 Homeopaths
		0370 Hospices
		0371 Hospitals
		0372 Mental health centres and practitioners
		0342 Midwifery
		0373 Nursing and residential care homes
		0344 Optometrists and opticians
		0809 Parenting and childcare services
		0345 Physical therapy
		0352 Speech therapists
		0354 Surgeons and cosmetic surgeries
		0812 Walk-in centre
		29 Health support services
		0356 Ambulance and medical transportation services
		0357 Blood transfusion service
		0358 Counselling and advice services
		0359 Health authorities
		0106 Medical equipment rental and leasing
		0361 Medical waste disposal services
		0362 Pregnancy related services and help centres
		0363 X-ray services
	31 Primary, secondary and tertiary education	0379 Broad age range and secondary state schools
		0375 First, primary and infant schools
		0376 Further education establishments
		0381 Higher education establishments
		0377 Independent and preparatory schools
		0801 Pupil referral units
		0380 Special schools and colleges
		0382 Unspecified and other schools
	32 Recreational and vocational education	0384 Ballet and dance schools
		0385 Beauty and hairdressing schools
		0388 Diving schools
		0389 Drama schools
		0390 Driving and motorcycle schools
		0391 First aid training
		0392 Flying schools
		0394 Language schools
		0395 Martial arts instruction
		0396 Music teachers and schools

		0397 Nursery schools and pre and after school care
		0399 Sailing schools
		0400 Sports and fitness coaching
		0403 Training providers and centres
**06 Public Infrastructure**	34 Infrastructure and facilities	0453 Allotments
		0454 Cemeteries and crematoria
		0456 Halls and community centres
		0457 Letter boxes
		0458 Libraries
		0459 Places of worship
	35 Organisations	0769 Community networks and projects
		0448 Institutes and professional organisations
		0449 Political parties and related organisations
		0450 Religious organisations
		0447 Sports clubs and associations
		0452 Youth Organisations
**09 Retail**	46 Clothing and accessories	0797 Baby and nursery equipment and children's clothes
		0656 Clothing
		0657 Footwear
		0659 Jewellery and fashion accessories
		0660 Lingerie and hosiery
	47 Food, drink and multi item retail	0671 Alcoholic drinks including off licences and
		wholesalers
		0661 Bakeries
		0662 Butchers
		0768 Cash and carry
		0663 Confectioners
		0699 Convenience stores and independent
		supermarkets
		0665 Delicatessens
		0666 Fishmongers
		0667 Frozen foods
		0668 Green and new age goods
		0669 Grocers, farm shops and pick your own
		0670 Herbs and spices
		0703 Livestock markets
		0705 Markets
		0672 Organic, health, gourmet and kosher foods
		0819 Supermarket chains
		0798 Tea and coffee merchants
		48 Household, office, leisure and garden
		0824 Adult shops
		0712 Art and antiques
		0674 Books and maps
		0693 Camping and caravanning
		0675 Carpets, rugs, soft furnishings and needlecraft
		0714 Charity shops
		0676 China and glassware
		0827 Comic bookshops
		0828 Computer shops
		0720 Computer supplies
		0677 Cosmetics, toiletries, perfumes and
		hairdressing supplies
		0678 Craft supplies
		0679 Cycles and accessories
		0700 Department stores
		0701 Discount stores
		0680 DIY and home improvement

		0721 Domestic appliances
		0722 Electrical goods and components
		0716 Florists
		0766 Fuel distributors and suppliers
		0682 Furniture
		0764 Garages, garden and portable buildings
		0683 Garden centres and nurseries
		0684 Garden machinery and furniture
		0685 General household goods
		0717 Gifts and cards
		0686 Hobby, sports and pastime products
		0687 Leather goods, luggage and travel accessories
		including handbags
		0688 Lighting
		0704 Mail order and catalogue stores
		0689 Music and video
		0690 Musical instruments
		0718 Party goods and novelties
		0691 Pets, supplies and services
		0724 Photographic and optical equipment
		0763 Post Offices
		0831 Potteries
		0719 Second hand goods
		0708 Shopping centres and retail parks
		0725 Stationery and office supplies
		0710 Surplus goods
		0726 Telephones and telephone cards
		0694 Travel agencies
	49 Motoring	0695 New vehicles
		0696 Second hand vehicles
		0697 Vehicle auctions
		0698 Vehicle parts and accessories
**10 Transport**	59 Bus transport	0732 Bus stops
		0759 Hail and ride zones
	57 Public transport, stations and infrastructure	0731 Bus and coach stations, depots and companies*
		0794 London Underground entrances
		0738 Railway stations, junctions and halts*
		0758 Taxi ranks
		0756 Tram, metro and light railway stations and stops
		0761 Underground network stations

* only those including the word "station" were retained.

Table S2: Baseline characteristics of UK Biobank participants (n = 57,726) by quintiles of *walkability*; all study participants resided within Greater London administrative boundaries at study baseline (2006-2010).

	**Walkability Quintiles**				
	**Q1 (N=11546)**	**Q2 (N=11545)**	**Q3 (N=11545)**	**Q4 (N=11545)**	**Q5 (N=11545)**
**Age at baseline (years)**					
Mean (SD)	57.5 (7.88)	56.4 (8.25)	55.7 (8.21)	55.2 (8.34)	55.0 (8.24)
**Sex**					
Female	6495 (56.3%)	6427 (55.7%)	6440 (55.8%)	6435 (55.7%)	6344 (55.0%)
Male	5051 (43.7%)	5118 (44.3%)	5105 (44.2%)	5110 (44.3%)	5201 (45.0%)
**Average total household income before tax (£)**					
Less than 18,000	1280 (11.1%)	1687 (14.6%)	1900 (16.5%)	1942 (16.8%)	2116 (18.3%)
18,000 to 30,999	2039 (17.7%)	2127 (18.4%)	2087 (18.1%)	1979 (17.1%)	1874 (16.2%)
31,000 to 51,999	2633 (22.8%)	2500 (21.7%)	2441 (21.1%)	2340 (20.3%)	2019 (17.5%)
52,000 to 100,000	2734 (23.7%)	2501 (21.7%)	2315 (20.1%)	2249 (19.5%)	2245 (19.4%)
Greater than 100,000	1017 (8.8%)	898 (7.8%)	1059 (9.2%)	1232 (10.7%)	1588 (13.8%)
Missing	1843 (16.0%)	1832 (15.9%)	1743 (15.1%)	1803 (15.6%)	1703 (14.8%)
**Index of Multiple Deprivation (small-area level)**					
Medium deprivation	3482 (30.2%)	4864 (42.1%)	6124 (53.0%)	5814 (50.4%)	4907 (42.5%)

Low deprivation	7350 (63.7%)	5097 (44.1%)	3193 (27.7%)	2193 (19.0%)	406 (3.5%)
High deprivation	714 (6.2%)	1584 (13.7%)	2228 (19.3%)	3538 (30.6%)	6232 (54.0%)
**IPAQ activity group**					
Low	1739 (15.1%)	1640 (14.2%)	1606 (13.9%)	1619 (14.0%)	1451 (12.6%)
Moderate	4132 (35.8%)	4080 (35.3%)	4151 (36.0%)	4263 (36.9%)	4385 (38.0%)
High	3714 (32.2%)	3813 (33.0%)	3762 (32.6%)	3786 (32.8%)	3859 (33.4%)
Missing	1961 (17.0%)	2012 (17.4%)	2026 (17.5%)	1877 (16.3%)	1850 (16.0%)
**Achieved UK physical activity recommendations via MVPA only**					
Yes	7865 (68.1%)	7898 (68.4%)	7939 (68.8%)	8103 (70.2%)	8245 (71.4%)
No	1717 (14.9%)	1633 (14.1%)	1576 (13.7%)	1563 (13.5%)	1447 (12.5%)
Missing	1964 (17.0%)	2014 (17.4%)	2030 (17.6%)	1879 (16.3%)	1853 (16.1%)
**Achieved UK physical activity recommendations via MVPA only**					
Yes	5196 (45.0%)	5206 (45.1%)	5163 (44.7%)	5169 (44.8%)	5241 (45.4%)
No	4389 (38.0%)	4327 (37.5%)	4356 (37.7%)	4499 (39.0%)	4454 (38.6%)
Missing	1961 (17.0%)	2012 (17.4%)	2026 (17.5%)	1877 (16.3%)	1850 (16.0%)
**Non-commuting transport**					
Car/motor vehicle	6893 (59.7%)	5809 (50.3%)	5041 (43.7%)	4351 (37.7%)	2990 (25.9%)
Cycle	116 (1.0%)	162 (1.4%)	193 (1.7%)	198 (1.7%)	348 (3.0%)
Walk	1251 (10.8%)	1874 (16.2%)	2349 (20.3%)	2860 (24.8%)	3597 (31.2%)
Public transport	760 (6.6%)	1116 (9.7%)	1380 (12.0%)	1680 (14.6%)	2030 (17.6%)
Missing	2526 (21.9%)	2584 (22.4%)	2582 (22.4%)	2456 (21.4%)	2580 (22.4%)
**Commuting transport**					
Car/motor vehicle	3049 (26.4%)	2837 (24.6%)	2542 (22.0%)	1994 (17.3%)	1300 (11.3%)
Cycle	104 (0.9%)	174 (1.5%)	188 (1.6%)	233 (2.0%)	395 (3.4%)
Walk	563 (4.9%)	700 (6.1%)	850 (7.4%)	1083 (9.4%)	1552 (13.4%)
Public transport	871 (7.5%)	1137 (9.8%)	1420 (12.3%)	1788 (15.5%)	1903 (16.5%)
Missing	6959 (60.3%)	6697 (58%)	6545 (56.7%)	6447 (55.5%)	6395 (55.4%)

Table S3: Baseline characteristics of UK Biobank participants (n = 57,726) by quintiles of *green walkability*; all study participants resided within Greater London administrative boundaries at study baseline (2006-2010).

	**Green walkability quintiles**				
	**Q1**	**Q2**	**Q3**	**Q4**	**Q5**
	**(N=11546)**	**(N=11545)**	**(N=11545)**	**(N=11545)**	**(N=11545)**
**Age at baseline (years)**					
Mean (SD)	57.2 (7.95)	56.1 (8.26)	55.8 (8.31)	55.6 (8.26)	55.2 (8.25)
**Sex**					
Female	6472 (56.1%)	6385 (55.3%)	6514 (56.4%)	6407 (55.5%)	6363 (55.1%)
Male	5074 (43.9%)	5160 (44.7%)	5031 (43.6%)	5138 (44.5%)	5182 (44.9%)
**Average total household income before tax (£)**					
Less than 18,000	1415 (12.3%)	1778 (15.4%)	1880 (16.3%)	1861 (16.1%)	1991 (17.2%)
18,000 to 30,999	2126 (18.4%)	2082 (18.0%)	2129 (18.4%)	1915 (16.6%)	1854 (16.1%)
31,000 to 51,999	2602 (22.5%)	2475 (21.4%)	2408 (20.9%)	2386 (20.7%)	2062 (17.9%)
52,000 to 100,000	2631 (22.8%)	2473 (21.4%)	2237 (19.4%)	2355 (20.4%)	2348 (20.3%)
Greater than 100,000	842 (7.3%)	875 (7.6%)	1130 (9.8%)	1321 (11.4%)	1626 (14.1%)
Missing	1930 (16.7%)	1862 (16.1%)	1761 (15.3%)	1707 (14.8%)	1664 (14.4%)
**Index of Multiple Deprivation (small-area level)**					
Medium deprivation	3845 (33.3%)	5358 (46.4%)	5590 (48.4%)	5374 (46.5%)	5024 (43.5%)
Low deprivation	6659 (57.7%)	4220 (36.6%)	3505 (30.4%)	3030 (26.2%)	825 (7.1%)
High deprivation	1042 (9.0%)	1967 (17.0%)	2450 (21.2%)	3141 (27.2%)	5696 (49.3%)
**IPAQ activity group**					
Low	1703 (14.7%)	1636 (14.2%)	1639 (14.2%)	1609 (13.9%)	1468 (12.7%)
Moderate	4090 (35.4%)	4152 (36.0%)	4135 (35.8%)	4173 (36.1%)	4461 (38.6%)
High	3705 (32.1%)	3686 (31.9%)	3822 (33.1%)	3899 (33.8%)	3822 (33.1%)
Missing	2048 (17.7%)	2071 (17.9%)	1949 (16.9%)	1864 (16.1%)	1794 (15.5%)
**Achieved UK physical activity recommendations via MVPA only**					
Yes	7827 (67.8%)	7845 (68.0%)	7972 (69.1%)	8110 (70.2%)	8296 (71.9%)
No	1667 (14.4%)	1627 (14.1%)	1622 (14.0%)	1567 (13.6%)	1453 (12.6%)
Missing	2052 (17.8%)	2073 (18.0%)	1951 (16.9%)	1868 (16.2%)	1796 (15.6%)
**Achieved UK physical activity recommendations via MVPA only**					
Yes	5147 (44.6%)	5094 (44.1%)	5202 (45.1%)	5254 (45.5%)	5278 (45.7%)
No	4351 (37.7%)	4380 (37.9%)	4394 (38.1%)	4427 (38.3%)	4473 (38.7%)
Missing	2048 (17.7%)	2071 (17.9%)	1949 (16.9%)	1864 (16.1%)	1794 (15.5%)

**Non-commuting transport**					
Car/motor vehicle	6724 (58.2%)	5637 (48.8%)	5006 (43.4%)	4422 (38.3%)	3295 (28.5%)
Cycle	90 (0.8%)	179 (1.6%)	187 (1.6%)	223 (1.9%)	338 (2.9%)
Walk	1388 (12.0%)	1900 (16.5%)	2380 (20.6%)	2826 (24.5%)	3437 (29.8%)
Public transport	819 (7.1%)	1184 (10.3%)	1464 (12.7%)	1556 (13.5%)	1943 (16.8%)
Missing	2525 (21.9%)	2645 (22.9%)	2508 (21.7%)	2518 (21.8%)	2532 (22%)
**Commuting transport**					
Car/motor vehicle	3021 (26.2%)	2799 (24.2%)	2416 (20.9%)	2013 (17.4%)	1473 (12.8%)
Cycle	103 (0.9%)	168 (1.5%)	192 (1.7%)	248 (2.1%)	383 (3.3%)
Walk	602 (5.2%)	711 (6.2%)	900 (7.8%)	1084 (9.4%)	1451 (12.6%)
Public transport	954 (8.3%)	1204 (10.4%)	1490 (12.9%)	1600 (13.9%)	1871 (16.2%)
Missing	6886 (59.5%)	6663 (57.8%)	6547 (56.7%)	6600 (57.2%)	6367 (55.2%)

Table S4: Baseline characteristics of UK Biobank participants (n = 57,726) by quintiles of *ground-cover walkability*; all study participants resided within Greater London administrative boundaries at study baseline (2006-2010).

	**Ground-cover walkability quintiles**				
	**Q1**	**Q2**	**Q3**	**Q4**	**Q5**
	**(N=11546)**	**(N=11545)**	**(N=11545)**	**(N=11545)**	**(N=11545)**
**Age at baseline (years)**					
Mean (SD)	57.6 (7.82)	56.0 (8.23)	55.8 (8.31)	55.4 (8.33)	55.1 (8.25)
**Sex**					
Female	6519 (56.5%)	6431 (55.7%)	6403 (55.5%)	6408 (55.5%)	6380 (55.3%)
Male	5027 (43.5%)	5114 (44.3%)	5142 (44.5%)	5137 (44.5%)	5165 (44.7%)
**Average total household income before tax (£)**					
Less than 18,000	1214 (10.5%)	1727 (15.0%)	1924 (16.7%)	2033 (17.6%)	2027 (17.6%)
18,000 to 30,999	1986 (17.2%)	2044 (17.7%)	2171 (18.8%)	2072 (17.9%)	1833 (15.9%)
31,000 to 51,999	2563 (22.2%)	2495 (21.6%)	2454 (21.3%)	2327 (20.2%)	2094 (18.1%)
52,000 to 100,000	2845 (24.6%)	2527 (21.9%)	2207 (19.1%)	2114 (18.3%)	2351 (20.4%)
Greater than 100,000	1132 (9.8%)	1045 (9.1%)	943 (8.2%)	1143 (9.9%)	1531 (13.3%)
Missing	1806 (15.6%)	1707 (14.8%)	1846 (16.0%)	1856 (16.1%)	1709 (14.8%)
**Index of Multiple Deprivation (small-area level)**					
Medium deprivation	3512 (30.4%)	5201 (45.0%)	5719 (49.5%)	5815 (50.4%)	4944 (42.8%)
Low deprivation	7379 (63.9%)	4512 (39.1%)	3213 (27.8%)	2082 (18.0%)	1053 (9.1%)
High deprivation	655 (5.7%)	1832 (15.9%)	2613 (22.6%)	3648 (31.6%)	5548 (48.1%)
**IPAQ activity group**					
Low	1667 (14.4%)	1655 (14.3%)	1602 (13.9%)	1661 (14.4%)	1470 (12.7%)
Moderate	4201 (36.4%)	4192 (36.3%)	4138 (35.8%)	4088 (35.4%)	4392 (38.0%)
High	3802 (32.9%)	3779 (32.7%)	3725 (32.3%)	3791 (32.8%)	3837 (33.2%)
Missing	1876 (16.2%)	1919 (16.6%)	2080 (18.0%)	2005 (17.4%)	1846 (16.0%)
**Achieved UK physical activity recommendations via MVPA only**					
Yes	8022 (69.5%)	7972 (69.1%)	7881 (68.3%)	7941 (68.8%)	8234 (71.3%)
No	1645 (14.2%)	1651 (14.3%)	1581 (13.7%)	1596 (13.8%)	1463 (12.7%)
Missing	1879 (16.3%)	1922 (16.6%)	2083 (18.0%)	2008 (17.4%)	1848 (16.0%)
**Achieved UK physical activity recommendations via MVPA only**					
Yes	5279 (45.7%)	5197 (45.0%)	5112 (44.3%)	5138 (44.5%)	5249 (45.5%)
No	4391 (38.0%)	4429 (38.4%)	4353 (37.7%)	4402 (38.1%)	4450 (38.5%)
Missing	1876 (16.2%)	1919 (16.6%)	2080 (18.0%)	2005 (17.4%)	1846 (16.0%)
**Non-commuting transport**					
Car/motor vehicle	6659 (57.7%)	5546 (48.0%)	5012 (43.4%)	4465 (38.7%)	3402 (29.5%)
Cycle	102 (0.9%)	199 (1.7%)	188 (1.6%)	211 (1.8%)	317 (2.7%)
Walk	1466 (12.7%)	2009 (17.4%)	2337 (20.2%)	2721 (23.6%)	3398 (29.4%)
Public transport	840 (7.3%)	1125 (9.7%)	1442 (12.5%)	1659 (14.4%)	1900 (16.5%)
Missing	2479 (21.4%)	2666 (23.1%)	2566 (22.2%)	2489 (21.6%)	2528 (22%)
**Commuting transport**					
Car/motor vehicle	2848 (24.7%)	2734 (23.7%)	2470 (21.4%)	2124 (18.4%)	1546 (13.4%)
Cycle	101 (0.9%)	197 (1.7%)	192 (1.7%)	239 (2.1%)	365 (3.2%)
Walk	633 (5.5%)	772 (6.7%)	882 (7.6%)	1039 (9.0%)	1422 (12.3%)
Public transport	969 (8.4%)	1234 (10.7%)	1454 (12.6%)	1621 (14.0%)	1841 (15.9%)
Missing	6995 (60.6%)	6608 (57.3%)	6547 (56.8%)	6522 (56.5%)	6371 (55.1%)

Table S5: Baseline characteristics of UK Biobank participants (n = 57,726) by quintiles of *tree-cover walkability*; all study participants resided within Greater London administrative boundaries at study baseline (2006-2010).

	**Tree-cover walkability quintiles**				
	**Q1**	**Q2**	**Q3**	**Q4**	**Q5**
	**(N=11546)**	**(N=11545)**	**(N=11545)**	**(N=11545)**	**(N=11545)**
**Age at baseline (years)**					
Mean (SD)	56.8 (8.16)	56.5 (8.11)	56.0 (8.31)	55.3 (8.26)	55.3 (8.23)
**Sex**					
Female	6378 (55.2%)	6441 (55.8%)	6544 (56.7%)	6428 (55.7%)	6350 (55.0%)
Male	5168 (44.8%)	5104 (44.2%)	5001 (43.3%)	5117 (44.3%)	5195 (45.0%)
**Average total household income before tax (£)**					
Less than 18,000	1717 (14.9%)	1725 (14.9%)	1638 (14.2%)	1836 (15.9%)	2009 (17.4%)
18,000 to 30,999	2204 (19.1%)	2154 (18.7%)	1979 (17.1%)	1950 (16.9%)	1819 (15.8%)
31,000 to 51,999	2591 (22.4%)	2520 (21.8%)	2474 (21.4%)	2279 (19.7%)	2069 (17.9%)
52,000 to 100,000	2364 (20.5%)	2423 (21.0%)	2489 (21.6%)	2457 (21.3%)	2311 (20.0%)
Greater than 100,000	641 (5.6%)	788 (6.8%)	1299 (11.3%)	1393 (12.1%)	1673 (14.5%)
Missing	2029 (17.6%)	1935 (16.8%)	1666 (14.4%)	1630 (14.1%)	1664 (14.4%)
**Index of Multiple Deprivation (small-area level)**					
Medium deprivation	4278 (37.1%)	5029 (43.6%)	5584 (48.4%)	5111 (44.3%)	5189 (44.9%)
Low deprivation	5633 (48.8%)	4796 (41.5%)	4012 (34.8%)	3270 (28.3%)	528 (4.6%)
High deprivation	1635 (14.2%)	1720 (14.9%)	1949 (16.9%)	3164 (27.4%)	5828 (50.5%)
**IPAQ activity group**					
Low	1719 (14.9%)	1692 (14.7%)	1598 (13.8%)	1604 (13.9%)	1442 (12.5%)
Moderate	3964 (34.3%)	4065 (35.2%)	4228 (36.6%)	4322 (37.4%)	4432 (38.4%)
High	3640 (31.5%)	3700 (32.0%)	3889 (33.7%)	3843 (33.3%)	3862 (33.5%)
Missing	2223 (19.3%)	2088 (18.1%)	1830 (15.9%)	1776 (15.4%)	1809 (15.7%)
**Achieved UK physical activity recommendations via MVPA only**					
Yes	7614 (65.9%)	7813 (67.7%)	8116 (70.3%)	8194 (71.0%)	8313 (72.0%)
No	1706 (14.8%)	1641 (14.2%)	1599 (13.9%)	1569 (13.6%)	1421 (12.3%)
Missing	2226 (19.3%)	2091 (18.1%)	1830 (15.9%)	1782 (15.4%)	1811 (15.7%)
**Achieved UK physical activity recommendations via MVPA only**					
Yes	5045 (43.7%)	5076 (44.0%)	5329 (46.2%)	5240 (45.4%)	5285 (45.8%)
No	4278 (37.1%)	4381 (37.9%)	4386 (38.0%)	4529 (39.2%)	4451 (38.6%)
Missing	2223 (19.3%)	2088 (18.1%)	1830 (15.9%)	1776 (15.4%)	1809 (15.7%)
**Non-commuting transport**					
Car/motor vehicle	6530 (56.6%)	5939 (51.4%)	5196 (45.0%)	4267 (37.0%)	3152 (27.3%)
Cycle	120 (1.0%)	127 (1.1%)	203 (1.8%)	232 (2.0%)	335 (2.9%)
Walk	1400 (12.1%)	1833 (15.9%)	2269 (19.7%)	2897 (25.1%)	3532 (30.6%)
Public transport	936 (8.1%)	1098 (9.5%)	1336 (11.6%)	1626 (14.1%)	1970 (17.1%)
Missing	2596 (22.2%)	2548 (22%)	2541 (22.1%)	2523 (21.8%)	2556 (22.2%)
**Commuting transport**					
Car/motor vehicle	3194 (27.7%)	2864 (24.8%)	2404 (20.8%)	1909 (16.5%)	1351 (11.7%)
Cycle	106 (0.9%)	148 (1.3%)	196 (1.7%)	263 (2.3%)	381 (3.3%)
Walk	553 (4.8%)	722 (6.3%)	820 (7.1%)	1141 (9.9%)	1512 (13.1%)
Public transport	951 (8.2%)	1119 (9.7%)	1454 (12.6%)	1730 (15.0%)	1865 (16.2%)
Missing	6742 (58.4%)	6692 (57.9%)	6671 (57.9%)	6502 (56.4%)	6436 (55.8%)

Table S6: Baseline characteristics of UK Biobank participants with 7-day accelerometer information (n = 12,986) by quintiles of *walkability*; all study participants resided within Greater London administrative boundaries at study baseline (2006-2010).

	**Walkability Quintiles**				
	**Q1**	**Q2**	**Q3**	**Q4**	**Q5**
	**(N=2598)**	**(N=2597)**	**(N=2597)**	**(N=2597)**	**(N=2597)**
**Age at baseline (years)**					
**Mean (SD)**	57.6 (7.59)	56.6 (8.04)	56.1 (7.82)	55.2 (8.26)	54.9 (8.01)
**Sex**					
**Female**	1513 (58.2%)	1512 (58.2%)	1488 (57.3%)	1571 (60.5%)	1588 (61.1%)
**Male**	1085 (41.8%)	1085 (41.8%)	1109 (42.7%)	1026 (39.5%)	1009 (38.9%)
**Average total household income before tax (£)**					
**Less than 18,000**	197 (7.6%)	269 (10.4%)	283 (10.9%)	276 (10.6%)	284 (10.9%)
**18,000 to 30,999**	423 (16.3%)	479 (18.4%)	434 (16.7%)	420 (16.2%)	406 (15.6%)

**31,000 to 51,999**	633 (24.4%)	627 (24.1%)	623 (24.0%)	629 (24.2%)	542 (20.9%)
**52,000 to 100,000**	736 (28.3%)	697 (26.8%)	676 (26.0%)	690 (26.6%)	681 (26.2%)
**Greater than 100,000**	309 (11.9%)	257 (9.9%)	324 (12.5%)	370 (14.2%)	488 (18.8%)
**Missing**	300 (11.5%)	268 (10.3%)	257 (9.9%)	212 (8.2%)	196 (7.5%)
**Index of Multiple Deprivation (small-area level)**					
**Low deprivation**	1788 (68.8%)	1318 (50.8%)	919 (35.4%)	670 (25.8%)	128 (4.9%)
**Medium deprivation**	717 (27.6%)	1043 (40.2%)	1326 (51.1%)	1378 (53.1%)	1276 (49.1%)
**High deprivation**	93 (3.6%)	236 (9.1%)	352 (13.6%)	549 (21.1%)	1193 (45.9%)
**IPAQ activity group**					
**Low**	383 (14.7%)	343 (13.2%)	337 (13.0%)	346 (13.3%)	304 (11.7%)
**Moderate**	979 (37.7%)	984 (37.9%)	1005 (38.7%)	1012 (39.0%)	1061 (40.9%)
**High**	891 (34.3%)	903 (34.8%)	927 (35.7%)	922 (35.5%)	951 (36.6%)
**Missing**	345 (13.3%)	367 (14.1%)	328 (12.6%)	317 (12.2%)	281 (10.8%)
**Achieved UK physical activity recommendations via MVPA and walking**					
**Yes**	1879 (72.3%)	1878 (72.3%)	1933 (74.4%)	1945 (74.9%)	2017 (77.7%)
**No**	374 (14.4%)	352 (13.6%)	336 (12.9%)	335 (12.9%)	299 (11.5%)
**Missing**	345 (13.3%)	367 (14.1%)	328 (12.6%)	317 (12.2%)	281 (10.8%)
**Achieved UK physical activity recommendations via MVPA only**					
**Yes**	1253 (48.2%)	1235 (47.6%)	1246 (48.0%)	1260 (48.5%)	1300 (50.1%)
**No**	1000 (38.5%)	995 (38.3%)	1023 (39.4%)	1020 (39.3%)	1016 (39.1%)
**Missing**	345 (13.3%)	367 (14.1%)	328 (12.6%)	317 (12.2%)	281 (10.8%)
**7-day accelerometer: minutes spent doing MVPA (≥ 125 mg)**					
**Mean (SD)**	73.5 (35.1)	74.9 (37.0)	75.3 (35.9)	77.4 (36.2)	78.1 (35.9)
**7-day accelerometer: minutes spent doing moderate activity (≥ 125 mg and <425 mg)**					
**Mean (SD)**	69.6 (32.4)	71.0 (34.0)	71.4 (33.4)	73.3 (33.5)	74.2 (33.4)
**7-day accelerometer: minutes spent doing vigorous activity (≥ 425 mg)**					
**Mean (SD)**	3.88 (5.32)	3.92 (5.82)	3.88 (5.54)	4.13 (5.83)	3.83 (5.57)
**Non-commuting transport**					
**Car/motor vehicle**	1601 (61.6%)	1325 (51.0%)	1137 (43.8%)	1000 (38.5%)	701 (27.0%)
**Cycle**	35 (1.3%)	48 (1.8%)	49 (1.9%)	60 (2.3%)	104 (4.0%)
**Walk**	264 (10.2%)	450 (17.3%)	587 (22.6%)	721 (27.8%)	907 (34.9%)
**Public transport**	155 (6.0%)	220 (8.5%)	217 (8.4%)	274 (10.6%)	294 (11.3%)
**Missing**	543 (20.9%)	554 (21.3%)	607 (23.3%)	542 (20.8%)	591 (22.8%)
**Commuting transport**					
**Car/motor vehicle**	677 (26.1%)	630 (24.3%)	550 (21.2%)	409 (15.7%)	310 (11.9%)
**Cycle**	31 (1.2%)	47 (1.8%)	43 (1.7%)	79 (3.0%)	126 (4.9%)
**Walk**	155 (6.0%)	197 (7.6%)	246 (9.5%)	304 (11.7%)	438 (16.9%)
**Public transport**	190 (7.3%)	278 (10.7%)	299 (11.5%)	393 (15.1%)	410 (15.8%)
**Missing**	1545 (59.4%)	1445 (55.7%)	1459 (56.1%)	1412 (54.3%)	1313 (50.5%)

Table S7: Baseline characteristics of UK Biobank participants with 7-day accelerometer information (n = 12,986) by quintiles of *green walkability*; all study participants resided within Greater London administrative boundaries at study baseline (2006-2010).

	**Green walkability quintiles**				
	**Q1**	**Q2**	**Q3**	**Q4**	**Q5**
	**(N=2598)**	**(N=2597)**	**(N=2597)**	**(N=2597)**	**(N=2597)**
**Age at baseline (years)**					
**Mean (SD)**	57.2 (7.64)	56.3 (8.05)	56.1 (8.09)	55.6 (8.04)	55.1 (8.05)
**Sex**					
**Female**	1531 (58.9%)	1471 (56.6%)	1533 (59.0%)	1539 (59.3%)	1598 (61.5%)
**Male**	1067 (41.1%)	1126 (43.4%)	1064 (41.0%)	1058 (40.7%)	999 (38.5%)
**Average total household income before tax (£)**					
**Less than 18,000**	218 (8.4%)	259 (10.0%)	284 (10.9%)	276 (10.6%)	272 (10.5%)
**18,000 to 30,999**	464 (17.9%)	468 (18.0%)	457 (17.6%)	373 (14.4%)	400 (15.4%)
**31,000 to 51,999**	633 (24.4%)	637 (24.5%)	591 (22.8%)	651 (25.1%)	542 (20.9%)
**52,000 to 100,000**	724 (27.9%)	696 (26.8%)	644 (24.8%)	715 (27.5%)	701 (27.0%)
**Greater than 100,000**	257 (9.9%)	282 (10.9%)	354 (13.6%)	366 (14.1%)	489 (18.8%)
**Missing**	302 (11.6%)	255 (9.8%)	267 (10.3%)	216 (8.3%)	193 (7.4%)

**Index of Multiple Deprivation (small-area level)**					
**Low deprivation**	1639 (63.1%)	1126 (43.4%)	953 (36.7%)	861 (33.2%)	244 (9.4%)
**Medium deprivation**	800 (30.8%)	1148 (44.2%)	1259 (48.5%)	1251 (48.2%)	1282 (49.4%)
**High deprivation**	159 (6.1%)	323 (12.4%)	385 (14.8%)	485 (18.7%)	1071 (41.2%)
**IPAQ activity group**					
**Low**	364 (14.0%)	372 (14.3%)	336 (12.9%)	337 (13.0%)	304 (11.7%)
**Moderate**	980 (37.7%)	997 (38.4%)	1012 (39.0%)	973 (37.5%)	1079 (41.5%)
**High**	886 (34.1%)	889 (34.2%)	926 (35.7%)	976 (37.6%)	917 (35.3%)
**Missing**	368 (14.2%)	339 (13.1%)	323 (12.4%)	311 (12.0%)	297 (11.4%)
**Achieved UK physical activity recommendations via MVPA and walking**					
**Yes**	1873 (72.1%)	1883 (72.5%)	1945 (74.9%)	1950 (75.1%)	2001 (77.1%)
**No**	357 (13.7%)	375 (14.4%)	329 (12.7%)	336 (12.9%)	299 (11.5%)
**Missing**	368 (14.2%)	339 (13.1%)	323 (12.4%)	311 (12.0%)	297 (11.4%)
**Achieved UK physical activity recommendations via MVPA only**					
**Yes**	1230 (47.3%)	1235 (47.6%)	1242 (47.8%)	1290 (49.7%)	1297 (49.9%)
**No**	1000 (38.5%)	1023 (39.4%)	1032 (39.7%)	996 (38.4%)	1003 (38.6%)
**Missing**	368 (14.2%)	339 (13.1%)	323 (12.4%)	311 (12.0%)	297 (11.4%)
**7-day accelerometer: minutes spent doing MVPA (≥ 125 mg)**					
**Mean (SD)**	73.2 (35.1)	75.1 (37.0)	75.6 (35.6)	77.7 (37.0)	77.6 (35.4)
**7-day accelerometer: minutes spent doing moderate activity (≥ 125 mg and <425 mg)**					
**Mean (SD)**	69.5 (32.6)	71.0 (34.0)	71.7 (32.9)	73.6 (34.2)	73.7 (32.9)
**7-day accelerometer: minutes spent doing vigorous activity (≥ 425 mg)**					
**Mean (SD)**	3.68 (4.90)	4.08 (6.02)	3.83 (5.51)	4.16 (5.97)	3.89 (5.61)
**Non-commuting transport**					
**Car/motor vehicle**	1535 (59.1%)	1297 (49.9%)	1139 (43.9%)	1014 (39.0%)	779 (30.0%)
**Cycle**	27 (1.0%)	48 (1.8%)	52 (2.0%)	77 (3.0%)	92 (3.5%)
**Walk**	313 (12.0%)	453 (17.4%)	595 (22.9%)	724 (27.9%)	844 (32.5%)
**Public transport**	166 (6.4%)	216 (8.3%)	238 (9.2%)	239 (9.2%)	301 (11.6%)
**Missing**	557 (21.4%)	583 (22.4%)	573 (22.1%)	543 (21%)	583 (22.3%)
**Commuting transport**					
**Car/motor vehicle**	673 (25.9%)	615 (23.7%)	520 (20.0%)	425 (16.4%)	343 (13.2%)
**Cycle**	25 (1.0%)	51 (2.0%)	50 (1.9%)	86 (3.3%)	114 (4.4%)
**Walk**	181 (7.0%)	187 (7.2%)	259 (10.0%)	308 (11.9%)	405 (15.6%)
**Public transport**	217 (8.4%)	287 (11.1%)	310 (11.9%)	349 (13.4%)	407 (15.7%)
**Missing**	1502 (57.8%)	1457 (56.3%)	1458 (56.2%)	1426 (55.0%)	1328 (51.1%)

Table S8: Baseline characteristics of UK Biobank participants with 7-day accelerometer information (n = 12,986) by quintiles of *ground-cover walkability*; all study participants resided within Greater London administrative boundaries at study baseline (2006-2010).

	**Ground-cover walkability quintiles**				
	**Q1**	**Q2**	**Q3**	**Q4**	**Q5**
	**(N=2598)**	**(N=2597)**	**(N=2597)**	**(N=2597)**	**(N=2597)**
**Age at baseline (years)**					
**Mean (SD)**	57.7 (7.57)	56.2 (7.99)	55.9 (8.05)	55.6 (8.11)	55.0 (8.08)
**Sex**					
**Female**	1515 (58.3%)	1509 (58.1%)	1507 (58.0%)	1551 (59.7%)	1590 (61.2%)
**Male**	1083 (41.7%)	1088 (41.9%)	1090 (42.0%)	1046 (40.3%)	1007 (38.8%)
**Average total household income before tax (£)**					
**Less than 18,000**	185 (7.1%)	273 (10.5%)	290 (11.2%)	292 (11.2%)	269 (10.4%)
**18,000 to 30,999**	441 (17.0%)	436 (16.8%)	454 (17.5%)	438 (16.9%)	393 (15.1%)
**31,000 to 51,999**	606 (23.3%)	609 (23.5%)	653 (25.1%)	629 (24.2%)	557 (21.4%)
**52,000 to 100,000**	759 (29.2%)	686 (26.4%)	661 (25.5%)	656 (25.3%)	718 (27.6%)
**Greater than 100,000**	317 (12.2%)	344 (13.2%)	295 (11.4%)	333 (12.8%)	459 (17.7%)
**Missing**	290 (11.2%)	249 (9.6%)	244 (9.4%)	249 (9.6%)	201 (7.7%)
**Index of Multiple Deprivation (small-area level)**					
**Low deprivation**	1766 (68.0%)	1182 (45.5%)	942 (36.3%)	595 (22.9%)	338 (13.0%)
**Medium deprivation**	748 (28.8%)	1129 (43.5%)	1246 (48.0%)	1387 (53.4%)	1230 (47.4%)
**High deprivation**	84 (3.2%)	286 (11.0%)	409 (15.7%)	615 (23.7%)	1029 (39.6%)
**IPAQ activity group**					
**Low**	365 (14.0%)	371 (14.3%)	320 (12.3%)	355 (13.7%)	302 (11.6%)
**Moderate**	987 (38.0%)	1012 (39.0%)	1012 (39.0%)	987 (38.0%)	1043 (40.2%)
**High**	907 (34.9%)	898 (34.6%)	915 (35.2%)	925 (35.6%)	949 (36.5%)
**Missing**	339 (13.0%)	316 (12.2%)	350 (13.5%)	330 (12.7%)	303 (11.7%)

**Achieved UK physical activity recommendations via MVPA and walking**					
**Yes**	1897 (73.0%)	1905 (73.4%)	1935 (74.5%)	1927 (74.2%)	1988 (76.5%)
**No**	362 (13.9%)	376 (14.5%)	312 (12.0%)	340 (13.1%)	306 (11.8%)
**Missing**	339 (13.0%)	316 (12.2%)	350 (13.5%)	330 (12.7%)	303 (11.7%)
**Achieved UK physical activity recommendations via MVPA only**					
**Yes**	1255 (48.3%)	1237 (47.6%)	1258 (48.4%)	1244 (47.9%)	1300 (50.1%)
**No**	1004 (38.6%)	1044 (40.2%)	989 (38.1%)	1023 (39.4%)	994 (38.3%)
**Missing**	339 (13.0%)	316 (12.2%)	350 (13.5%)	330 (12.7%)	303 (11.7%)
**7-day accelerometer: minutes spent doing MVPA (≥ 125 mg)**					
**Mean (SD)**	74.0 (35.3)	75.9 (36.8)	75.4 (36.4)	76.1 (36.1)	77.8 (35.6)
**7-day accelerometer: minutes spent doing moderate activity (≥ 125 mg and <425 mg)**					
**Mean (SD)**	70.2 (32.7)	71.9 (33.9)	71.6 (33.8)	72.0 (33.4)	73.9 (33.0)
**7-day accelerometer: minutes spent doing vigorous activity (≥ 425 mg)**					
**Mean (SD)**	3.83 (5.31)	4.04 (6.13)	3.84 (5.37)	4.02 (5.57)	3.91 (5.67)
**Non-commuting transport**					
**Car/motor vehicle**	1532 (59.0%)	1238 (47.7%)	1163 (44.8%)	1049 (40.4%)	782 (30.1%)
**Cycle**	29 (1.1%)	52 (2.0%)	53 (2.0%)	72 (2.8%)	90 (3.5%)
**Walk**	325 (12.5%)	487 (18.8%)	572 (22.0%)	696 (26.8%)	849 (32.7%)
**Public transport**	163 (6.3%)	221 (8.5%)	230 (8.9%)	259 (10.0%)	287 (11.1%)
**Missing**	549 (21.1%)	599 (23.0%)	579 (22.3%)	516 (20.1%)	589 (22.7%)
**Commuting transport**					
**Car/motor vehicle**	632 (24.3%)	594 (22.9%)	535 (20.6%)	466 (17.9%)	349 (13.4%)
**Cycle**	21 (0.8%)	51 (2.0%)	56 (2.2%)	84 (3.2%)	114 (4.4%)
**Walk**	177 (6.8%)	200 (7.7%)	264 (10.2%)	291 (11.2%)	408 (15.7%)
**Public transport**	214 (8.2%)	288 (11.1%)	314 (12.1%)	340 (13.1%)	414 (15.9%)
**Missing**	1554 (59.8%)	1464 (56.3%)	1428 (54.9%)	1416 (54.5%)	1312 (50.5%)

Table S9: Baseline characteristics of UK Biobank participants with 7-day accelerometer information (n = 12,986) by quintiles of *tree-cover walkability*; all study participants resided within Greater London administrative boundaries at study baseline (2006-2010).

	**Ground-cover walkability quintiles**				
	**Q1**	**Q2**	**Q3**	**Q4**	**Q5**
	**(N=2598)**	**(N=2597)**	**(N=2597)**	**(N=2597)**	**(N=2597)**
**Age at baseline (years)**					
**Mean (SD)**	57.7 (7.57)	56.2 (7.99)	55.9 (8.05)	55.6 (8.11)	55.0 (8.08)
**Sex**					
**Female**	1515 (58.3%)	1509 (58.1%)	1507 (58.0%)	1551 (59.7%)	1590 (61.2%)
**Male**	1083 (41.7%)	1088 (41.9%)	1090 (42.0%)	1046 (40.3%)	1007 (38.8%)
**Average total household income before tax (£)**					
**Less than 18,000**	185 (7.1%)	273 (10.5%)	290 (11.2%)	292 (11.2%)	269 (10.4%)
**18,000 to 30,999**	441 (17.0%)	436 (16.8%)	454 (17.5%)	438 (16.9%)	393 (15.1%)
**31,000 to 51,999**	606 (23.3%)	609 (23.5%)	653 (25.1%)	629 (24.2%)	557 (21.4%)
**52,000 to 100,000**	759 (29.2%)	686 (26.4%)	661 (25.5%)	656 (25.3%)	718 (27.6%)
**Greater than 100,000**	317 (12.2%)	344 (13.2%)	295 (11.4%)	333 (12.8%)	459 (17.7%)
**Missing**	290 (11.2%)	249 (9.6%)	244 (9.4%)	249 (9.6%)	201 (7.7%)
**Index of Multiple Deprivation (small-area level)**					
**Low deprivation**	1766 (68.0%)	1182 (45.5%)	942 (36.3%)	595 (22.9%)	338 (13.0%)
**Medium deprivation**	748 (28.8%)	1129 (43.5%)	1246 (48.0%)	1387 (53.4%)	1230 (47.4%)
**High deprivation**	84 (3.2%)	286 (11.0%)	409 (15.7%)	615 (23.7%)	1029 (39.6%)
**IPAQ activity group**					
**Low**	365 (14.0%)	371 (14.3%)	320 (12.3%)	355 (13.7%)	302 (11.6%)
**Moderate**	987 (38.0%)	1012 (39.0%)	1012 (39.0%)	987 (38.0%)	1043 (40.2%)
**High**	907 (34.9%)	898 (34.6%)	915 (35.2%)	925 (35.6%)	949 (36.5%)
**Missing**	339 (13.0%)	316 (12.2%)	350 (13.5%)	330 (12.7%)	303 (11.7%)
**Achieved UK physical activity recommendations via MVPA and walking**[1]					
**Yes**	1897 (73.0%)	1905 (73.4%)	1935 (74.5%)	1927 (74.2%)	1988 (76.5%)
**No**	362 (13.9%)	376 (14.5%)	312 (12.0%)	340 (13.1%)	306 (11.8%)
**Missing**	339 (13.0%)	316 (12.2%)	350 (13.5%)	330 (12.7%)	303 (11.7%)
**Achieved UK physical activity recommendations via MVPA only**[1]					
**Yes**	1255 (48.3%)	1237 (47.6%)	1258 (48.4%)	1244 (47.9%)	1300 (50.1%)
**No**	1004 (38.6%)	1044 (40.2%)	989 (38.1%)	1023 (39.4%)	994 (38.3%)
**Missing**	339 (13.0%)	316 (12.2%)	350 (13.5%)	330 (12.7%)	303 (11.7%)
**7-day accelerometer: minutes spent doing MVPA (≥ 125 mg)**					

**Mean (SD)**	74.0 (35.3)	75.9 (36.8)	75.4 (36.4)	76.1 (36.1)	77.8 (35.6)
**7-day accelerometer: minutes spent doing moderate activity (≥ 125 mg and <425 mg)**					
**Mean (SD)**	70.2 (32.7)	71.9 (33.9)	71.6 (33.8)	72.0 (33.4)	73.9 (33.0)
**7-day accelerometer: minutes spent doing vigorous activity (≥ 425 mg)**					
**Mean (SD)**	3.83 (5.31)	4.04 (6.13)	3.84 (5.37)	4.02 (5.57)	3.91 (5.67)
**Non-commuting transport**					
**Car/motor vehicle**	1532 (59.0%)	1238 (47.7%)	1163 (44.8%)	1049 (40.4%)	782 (30.1%)
**Cycle**	29 (1.1%)	52 (2.0%)	53 (2.0%)	72 (2.8%)	90 (3.5%)
**Walk**	325 (12.5%)	487 (18.8%)	572 (22.0%)	696 (26.8%)	849 (32.7%)
**Public transport**	163 (6.3%)	221 (8.5%)	230 (8.9%)	259 (10.0%)	287 (11.1%)
**Missing**	549 (21.1%)	599 (23.0%)	579 (22.3%)	516 (20.1%)	589 (22.7%)
**Commuting transport**					
**Car/motor vehicle**	632 (24.3%)	594 (22.9%)	535 (20.6%)	466 (17.9%)	349 (13.4%)
**Cycle**	21 (0.8%)	51 (2.0%)	56 (2.2%)	84 (3.2%)	114 (4.4%)
**Walk**	177 (6.8%)	200 (7.7%)	264 (10.2%)	291 (11.2%)	408 (15.7%)
**Public transport**	214 (8.2%)	288 (11.1%)	314 (12.1%)	340 (13.1%)	414 (15.9%)
**Missing**	1554 (59.8%)	1464 (56.3%)	1428 (54.9%)	1416 (54.5%)	1312 (50.5%)

Table S10: Baseline characteristics of UK Biobank participants with non-commuting transport information (n = 44,998) by quintiles of *walkability*; all study participants resided within Greater London administrative boundaries at study baseline (2006-2010).

	**Walkability Quintiles**				
	**Q1**	**Q2**	**Q3**	**Q4**	**Q5**
	**(N=9021)**	**(N=8994)**	**(N=8998)**	**(N=8988)**	**(N=8997)**
**Age at baseline (years)**					
Mean (SD)	57.6 (7.87)	56.5 (8.26)	55.7 (8.24)	55.2 (8.36)	55.0 (8.27)
**Sex**					
Female	5040 (55.9%)	5008 (55.7%)	4991 (55.5%)	5032 (56.0%)	4944 (55.0%)
Male	3981 (44.1%)	3986 (44.3%)	4007 (44.5%)	3956 (44.0%)	4053 (45.0%)
**Average total household income before tax (£)**					
Less than 18,000	975 (10.8%)	1339 (14.9%)	1452 (16.1%)	1508 (16.8%)	1633 (18.2%)
18,000 to 30,999	1606 (17.8%)	1648 (18.3%)	1621 (18.0%)	1556 (17.3%)	1460 (16.2%)
31,000 to 51,999	2092 (23.2%)	1926 (21.4%)	1941 (21.6%)	1829 (20.3%)	1604 (17.8%)
52,000 to 100,000	2134 (23.7%)	1946 (21.6%)	1775 (19.7%)	1752 (19.5%)	1744 (19.4%)
Greater than 100,000	791 (8.8%)	701 (7.8%)	824 (9.2%)	952 (10.6%)	1232 (13.7%)
Missing	1423 (15.8%)	1434 (15.9%)	1385 (15.4%)	1391 (15.5%)	1324 (14.7%)
**Index of Multiple Deprivation (small-area level)**					
Medium deprivation	544 (6.0%)	1228 (13.7%)	1735 (19.3%)	2744 (30.5%)	4909 (54.6%)
Low deprivation	2707 (30.0%)	3778 (42.0%)	4789 (53.2%)	4542 (50.5%)	3766 (41.9%)
High deprivation	5770 (64.0%)	3988 (44.3%)	2474 (27.5%)	1702 (18.9%)	322 (3.6%)
**IPAQ activity group**					
Low	1356 (15.0%)	1265 (14.1%)	1239 (13.8%)	1248 (13.9%)	1121 (12.5%)
Moderate	3259 (36.1%)	3189 (35.5%)	3282 (36.5%)	3336 (37.1%)	3442 (38.3%)
High	2882 (31.9%)	2977 (33.1%)	2907 (32.3%)	2955 (32.9%)	3023 (33.6%)
Missing	1524 (16.9%)	1563 (17.4%)	1570 (17.4%)	1449 (16.1%)	1411 (15.7%)
**Achieved UK physical activity recommendations via MVPA only**					
Yes	6159 (68.3%)	6171 (68.6%)	6194 (68.8%)	6342 (70.6%)	6464 (71.8%)
No	1335 (14.8%)	1258 (14.0%)	1230 (13.7%)	1196 (13.3%)	1119 (12.4%)
Missing	1527 (16.9%)	1565 (17.4%)	1574 (17.5%)	1450 (16.1%)	1414 (15.7%)
**Achieved UK physical activity recommendations via MVPA only**					
Yes	4007 (44.4%)	4066 (45.2%)	4030 (44.8%)	4032 (44.9%)	4087 (45.4%)
No	3490 (38.7%)	3365 (37.4%)	3398 (37.8%)	3507 (39.0%)	3499 (38.9%)
Missing	1524 (16.9%)	1563 (17.4%)	1570 (17.4%)	1449 (16.1%)	1411 (15.7%)
**Non-commuting transport**					
Car/motor vehicle	6894 (76.4%)	5833 (64.9%)	5052 (56.1%)	4302 (47.9%)	3003 (33.4%)
Cycle	116 (1.3%)	162 (1.8%)	193 (2.1%)	198 (2.2%)	348 (3.9%)
Walk	1251 (13.9%)	1881 (20.9%)	2366 (26.3%)	2823 (31.4%)	3610 (40.1%)
Public transport	760 (8.4%)	1118 (12.4%)	1387 (15.4%)	1665 (18.5%)	2036 (22.6%)
**Commuting transport**					
Car/motor vehicle	3041 (33.7%)	2836 (31.5%)	2535 (28.2%)	1966 (21.9%)	1301 (14.5%)
Cycle	104 (1.2%)	174 (1.9%)	190 (2.1%)	229 (2.5%)	396 (4.4%)
Walk	563 (6.2%)	701 (7.8%)	855 (9.5%)	1063 (11.8%)	1558 (17.3%)

Public transport	870 (9.6%)	1139 (12.7%)	1425 (15.8%)	1758 (19.6%)	1906 (21.2%)
Missing	4443 (49.3%)	4144 (46.1%)	3993 (44.4%)	3972 (44.2%)	3836 (42.6%)

Table S11: Baseline characteristics of UK Biobank participants with non-commuting transport information (n = 44,998) by quintiles of *green walkability*; all study participants resided within Greater London administrative boundaries at study baseline (2006-2010).

	**Green walkability quintiles**				
	**Q1**	**Q2**	**Q3**	**Q4**	**Q5**
	**(N=9012)**	**(N=8988)**	**(N=9003)**	**(N=8995)**	**(N=9000)**
**Age at baseline (years)**					
Mean (SD)	57.3 (7.94)	56.1 (8.27)	55.8 (8.36)	55.6 (8.25)	55.1 (8.29)
**Sex**					
Female	5050 (56.0%)	4962 (55.2%)	5054 (56.1%)	4998 (55.6%)	4951 (55.0%)
Male	3962 (44.0%)	4026 (44.8%)	3949 (43.9%)	3997 (44.4%)	4049 (45.0%)
**Average total household income before tax (£)**					
Less than 18,000	1091 (12.1%)	1385 (15.4%)	1473 (16.4%)	1418 (15.8%)	1540 (17.1%)
18,000 to 30,999	1660 (18.4%)	1630 (18.1%)	1674 (18.6%)	1479 (16.4%)	1448 (16.1%)
31,000 to 51,999	2065 (22.9%)	1937 (21.6%)	1869 (20.8%)	1882 (20.9%)	1639 (18.2%)
52,000 to 100,000	2054 (22.8%)	1921 (21.4%)	1718 (19.1%)	1826 (20.3%)	1832 (20.4%)
Greater than 100,000	644 (7.1%)	665 (7.4%)	889 (9.9%)	1046 (11.6%)	1256 (14.0%)
Missing	1498 (16.6%)	1450 (16.1%)	1380 (15.3%)	1344 (14.9%)	1285 (14.3%)
**Index of Multiple Deprivation (small-area level)**					
Medium deprivation	789 (8.8%)	1545 (17.2%)	1918 (21.3%)	2421 (26.9%)	4487 (49.9%)
Low deprivation	2990 (33.2%)	4147 (46.1%)	4365 (48.5%)	4222 (46.9%)	3858 (42.9%)
High deprivation	5233 (58.1%)	3296 (36.7%)	2720 (30.2%)	2352 (26.1%)	655 (7.3%)
**IPAQ activity group**					
Low	1329 (14.7%)	1282 (14.3%)	1241 (13.8%)	1259 (14.0%)	1118 (12.4%)
Moderate	3197 (35.5%)	3275 (36.4%)	3284 (36.5%)	3225 (35.9%)	3527 (39.2%)
High	2890 (32.1%)	2827 (31.5%)	2955 (32.8%)	3069 (34.1%)	3003 (33.4%)
Missing	1596 (17.7%)	1604 (17.8%)	1523 (16.9%)	1442 (16.0%)	1352 (15.0%)
**Achieved UK physical activity recommendations via MVPA only**					
Yes	6117 (67.9%)	6101 (67.9%)	6243 (69.3%)	6337 (70.5%)	6532 (72.6%)
No	1295 (14.4%)	1281 (14.3%)	1235 (13.7%)	1213 (13.5%)	1114 (12.4%)
Missing	1600 (17.8%)	1606 (17.9%)	1525 (16.9%)	1445 (16.1%)	1354 (15.0%)
**Achieved UK physical activity recommendations via MVPA only**					
Yes	3993 (44.3%)	3914 (43.5%)	4052 (45.0%)	4124 (45.8%)	4139 (46.0%)
No	3423 (38.0%)	3470 (38.6%)	3428 (38.1%)	3429 (38.1%)	3509 (39.0%)
Missing	1596 (17.7%)	1604 (17.8%)	1523 (16.9%)	1442 (16.0%)	1352 (15.0%)
**Non-commuting transport**					
Car/motor vehicle	6720 (74.6%)	5687 (63.3%)	4982 (55.3%)	4405 (49.0%)	3290 (36.6%)
Cycle	90 (1.0%)	180 (2.0%)	187 (2.1%)	222 (2.5%)	338 (3.8%)
Walk	1387 (15.4%)	1918 (21.3%)	2377 (26.4%)	2816 (31.3%)	3433 (38.1%)
Public transport	815 (9.0%)	1203 (13.4%)	1457 (16.2%)	1552 (17.3%)	1939 (21.5%)
**Commuting transport**					
Car/motor vehicle	3006 (33.4%)	2818 (31.4%)	2392 (26.6%)	1998 (22.2%)	1465 (16.3%)
Cycle	103 (1.1%)	168 (1.9%)	197 (2.2%)	244 (2.7%)	381 (4.2%)
Walk	602 (6.7%)	710 (7.9%)	899 (10.0%)	1078 (12.0%)	1451 (16.1%)
Public transport	951 (10.6%)	1211 (13.5%)	1480 (16.4%)	1593 (17.7%)	1863 (20.7%)
Missing	4350 (48.3%)	4081 (45.4%)	4035 (44.8%)	4082 (45.4%)	3840 (42.7%)

Table S12: Baseline characteristics of UK Biobank participants with non-commuting transport information (n = 44,998) by quintiles of *ground-cover walkability*; all study participants resided within Greater London administrative boundaries at study baseline (2006-2010).

	**Ground-cover walkability quintiles**				
	**Q1**	**Q2**	**Q3**	**Q4**	**Q5**
	**(N=9023)**	**(N=8992)**	**(N=8989)**	**(N=8997)**	**(N=8997)**
**Age at baseline (years)**					
Mean (SD)	57.7 (7.82)	56.0 (8.25)	55.7 (8.31)	55.4 (8.34)	55.0 (8.30)
**Sex**					
Female	5070 (56.2%)	4998 (55.6%)	4961 (55.2%)	5003 (55.6%)	4983 (55.4%)
Male	3953 (43.8%)	3994 (44.4%)	4028 (44.8%)	3994 (44.4%)	4014 (44.6%)
**Average total household income before tax (£)**					

Less than 18,000	929 (10.3%)	1341 (14.9%)	1509 (16.8%)	1558 (17.3%)	1570 (17.5%)
18,000 to 30,999	1563 (17.3%)	1602 (17.8%)	1702 (18.9%)	1600 (17.8%)	1424 (15.8%)
31,000 to 51,999	2030 (22.5%)	1958 (21.8%)	1908 (21.2%)	1836 (20.4%)	1660 (18.5%)
52,000 to 100,000	2223 (24.6%)	1947 (21.7%)	1694 (18.8%)	1671 (18.6%)	1816 (20.2%)
Greater than 100,000	875 (9.7%)	810 (9.0%)	750 (8.3%)	877 (9.7%)	1188 (13.2%)
Missing	1403 (15.5%)	1334 (14.8%)	1426 (15.9%)	1455 (16.2%)	1339 (14.9%)
**Index of Multiple Deprivation (small-area level)**					
Medium deprivation	486 (5.4%)	1430 (15.9%)	2051 (22.8%)	2821 (31.4%)	4372 (48.6%)
Low deprivation	2724 (30.2%)	4019 (44.7%)	4478 (49.8%)	4580 (50.9%)	3781 (42.0%)
High deprivation	5813 (64.4%)	3543 (39.4%)	2460 (27.4%)	1596 (17.7%)	844 (9.4%)
**IPAQ activity group**					
Low	1302 (14.4%)	1275 (14.2%)	1254 (14.0%)	1269 (14.1%)	1129 (12.5%)
Moderate	3318 (36.8%)	3298 (36.7%)	3263 (36.3%)	3169 (35.2%)	3460 (38.5%)
High	2958 (32.8%)	2904 (32.3%)	2872 (32.0%)	3001 (33.4%)	3009 (33.4%)
Missing	1445 (16.0%)	1515 (16.8%)	1600 (17.8%)	1558 (17.3%)	1399 (15.5%)
**Achieved UK physical activity recommendations via MVPA only**					
Yes	6300 (69.8%)	6198 (68.9%)	6139 (68.3%)	6231 (69.3%)	6462 (71.8%)
No	1275 (14.1%)	1276 (14.2%)	1247 (13.9%)	1206 (13.4%)	1134 (12.6%)
Missing	1448 (16.0%)	1518 (16.9%)	1603 (17.8%)	1560 (17.3%)	1401 (15.6%)
**Achieved UK physical activity recommendations via MVPA only**					
Yes	4087 (45.3%)	4014 (44.6%)	3960 (44.1%)	4046 (45.0%)	4115 (45.7%)
No	3491 (38.7%)	3463 (38.5%)	3429 (38.1%)	3393 (37.7%)	3483 (38.7%)
Missing	1445 (16.0%)	1515 (16.8%)	1600 (17.8%)	1558 (17.3%)	1399 (15.5%)
**Non-commuting transport**					
Car/motor vehicle	6626 (73.4%)	5620 (62.5%)	5017 (55.8%)	4426 (49.2%)	3395 (37.7%)
Cycle	101 (1.1%)	201 (2.2%)	189 (2.1%)	210 (2.3%)	316 (3.5%)
Walk	1459 (16.2%)	2033 (22.6%)	2342 (26.1%)	2707 (30.1%)	3390 (37.7%)
Public transport	837 (9.3%)	1138 (12.7%)	1441 (16.0%)	1654 (18.4%)	1896 (21.1%)
**Commuting transport**					
Car/motor vehicle	2828 (31.3%)	2748 (30.6%)	2464 (27.4%)	2101 (23.4%)	1538 (17.1%)
Cycle	101 (1.1%)	199 (2.2%)	192 (2.1%)	238 (2.6%)	363 (4.0%)
Walk	630 (7.0%)	778 (8.7%)	883 (9.8%)	1031 (11.5%)	1418 (15.8%)
Public transport	959 (10.6%)	1248 (13.9%)	1449 (16.1%)	1609 (17.9%)	1833 (20.4%)
Missing	4505 (49.9%)	4019 (44.7%)	4001 (44.5%)	4018 (44.7%)	3845 (42.7%)

Table S13: Baseline characteristics of UK Biobank participants with non-commuting transport information (n = 44,998) by quintiles of *tree-cover walkability*; all study participants resided within Greater London administrative boundaries at study baseline (2006-2010).

	**Tree-cover walkability quintiles**				
	**Q1**	**Q2**	**Q3**	**Q4**	**Q5**
	**(N=9003)**	**(N=8996)**	**(N=8996)**	**(N=9005)**	**(N=8998)**
**Age at baseline (years)**					
Mean (SD)	56.8 (8.15)	56.5 (8.13)	55.9 (8.36)	55.3 (8.25)	55.3 (8.27)
**Sex**					
Female	4976 (55.3%)	5014 (55.7%)	5100 (56.7%)	4989 (55.4%)	4936 (54.9%)
Male	4027 (44.7%)	3982 (44.3%)	3896 (43.3%)	4016 (44.6%)	4062 (45.1%)
**Average total household income before tax (£)**					
Less than 18,000	1321 (14.7%)	1336 (14.9%)	1280 (14.2%)	1412 (15.7%)	1558 (17.3%)
18,000 to 30,999	1730 (19.2%)	1660 (18.5%)	1550 (17.2%)	1540 (17.1%)	1411 (15.7%)
31,000 to 51,999	2039 (22.6%)	1972 (21.9%)	1936 (21.5%)	1817 (20.2%)	1628 (18.1%)
52,000 to 100,000	1840 (20.4%)	1896 (21.1%)	1914 (21.3%)	1892 (21.0%)	1809 (20.1%)
Greater than 100,000	494 (5.5%)	617 (6.9%)	1011 (11.2%)	1077 (12.0%)	1301 (14.5%)
Missing	1579 (17.5%)	1515 (16.8%)	1305 (14.5%)	1267 (14.1%)	1291 (14.3%)
**Index of Multiple Deprivation (small-area level)**					
Medium deprivation	1256 (14.0%)	1338 (14.9%)	1520 (16.9%)	2461 (27.3%)	4585 (51.0%)
Low deprivation	3330 (37.0%)	3927 (43.7%)	4318 (48.0%)	4020 (44.6%)	3987 (44.3%)
High deprivation	4417 (49.1%)	3731 (41.5%)	3158 (35.1%)	2524 (28.0%)	426 (4.7%)
**IPAQ activity group**					
Low	1345 (14.9%)	1309 (14.6%)	1218 (13.5%)	1247 (13.8%)	1110 (12.3%)
Moderate	3096 (34.4%)	3195 (35.5%)	3324 (36.9%)	3407 (37.8%)	3486 (38.7%)
High	2835 (31.5%)	2870 (31.9%)	3019 (33.6%)	3001 (33.3%)	3019 (33.6%)
Missing	1727 (19.2%)	1622 (18.0%)	1435 (16.0%)	1350 (15.0%)	1383 (15.4%)
**Achieved UK physical activity recommendations via MVPA only**					

Yes	5937 (65.9%)	6096 (67.8%)	6343 (70.5%)	6438 (71.5%)	6516 (72.4%)
No	1336 (14.8%)	1275 (14.2%)	1218 (13.5%)	1212 (13.5%)	1097 (12.2%)
Missing	1730 (19.2%)	1625 (18.1%)	1435 (16.0%)	1355 (15.0%)	1385 (15.4%)
**Achieved UK physical activity recommendations via MVPA only**					
Yes	3909 (43.4%)	3949 (43.9%)	4131 (45.9%)	4113 (45.7%)	4120 (45.8%)
No	3367 (37.4%)	3425 (38.1%)	3430 (38.1%)	3542 (39.3%)	3495 (38.8%)
Missing	1727 (19.2%)	1622 (18.0%)	1435 (16.0%)	1350 (15.0%)	1383 (15.4%)
**Non-commuting transport**					
Car/motor vehicle	6542 (72.7%)	5939 (66.0%)	5187 (57.7%)	4262 (47.3%)	3154 (35.1%)
Cycle	120 (1.3%)	127 (1.4%)	203 (2.3%)	232 (2.6%)	335 (3.7%)
Walk	1405 (15.6%)	1830 (20.3%)	2270 (25.2%)	2889 (32.1%)	3537 (39.3%)
Public transport	936 (10.4%)	1100 (12.2%)	1336 (14.9%)	1622 (18.0%)	1972 (21.9%)
**Commuting transport**					
Car/motor vehicle	3189 (35.4%)	2847 (31.6%)	2392 (26.6%)	1903 (21.1%)	1348 (15.0%)
Cycle	106 (1.2%)	149 (1.7%)	195 (2.2%)	262 (2.9%)	381 (4.2%)
Walk	554 (6.2%)	721 (8.0%)	816 (9.1%)	1137 (12.6%)	1512 (16.8%)
Public transport	950 (10.6%)	1115 (12.4%)	1448 (16.1%)	1723 (19.1%)	1862 (20.7%)
Missing	4204 (46.7%)	4164 (46.3%)	4145 (46.1%)	3980 (44.2%)	3895 (43.3%)

Table S14: Baseline characteristics of UK Biobank participants with commuting transport information and who commuted at least once per week (n = 23,999) by quintiles of *walkability*; all study participants resided within Greater London administrative boundaries at study baseline (2006-2010).

	**Walkability Quintiles**				
	**Q1 (N=4804)**	**Q2 (N=4801)**	**Q3 (N=4798)**	**Q4 (N=4797)**	**Q5 (N=4799)**
**Age at baseline (years)**					
Mean (SD)	53.9 (7.24)	52.9 (7.37)	52.4 (7.24)	52.0 (7.26)	52.5 (7.45)
**Sex**					
Female	2597 (54.1%)	2566 (53.4%)	2575 (53.7%)	2575 (53.7%)	2612 (54.4%)
Male	2207 (45.9%)	2235 (46.6%)	2223 (46.3%)	2222 (46.3%)	2187 (45.6%)
**Average total household income before tax (£)**					
Less than 18,000	241 (5.0%)	367 (7.6%)	417 (8.7%)	373 (7.8%)	432 (9.0%)
18,000 to 30,999	666 (13.9%)	786 (16.4%)	840 (17.5%)	862 (18.0%)	831 (17.3%)
31,000 to 51,999	1244 (25.9%)	1214 (25.3%)	1256 (26.2%)	1166 (24.3%)	1082 (22.5%)
52,000 to 100,000	1564 (32.6%)	1401 (29.2%)	1243 (25.9%)	1231 (25.7%)	1204 (25.1%)
Greater than 100,000	584 (12.2%)	504 (10.5%)	567 (11.8%)	698 (14.6%)	866 (18.0%)
Missing	505 (10.5%)	529 (11.0%)	475 (9.9%)	467 (9.7%)	384 (8.0%)
**Index of Multiple Deprivation (small-area level)**					
Low deprivation	319 (6.6%)	689 (14.4%)	986 (20.6%)	1567 (32.7%)	2580 (53.8%)
Medium deprivation	1505 (31.3%)	2104 (43.8%)	2617 (54.5%)	2374 (49.5%)	2067 (43.1%)
High deprivation	2980 (62.0%)	2008 (41.8%)	1195 (24.9%)	856 (17.8%)	152 (3.2%)
**IPAQ activity group**					
Low	807 (16.8%)	704 (14.7%)	669 (13.9%)	683 (14.2%)	556 (11.6%)
Moderate	1792 (37.3%)	1754 (36.5%)	1775 (37.0%)	1835 (38.3%)	1903 (39.7%)
High	1477 (30.7%)	1613 (33.6%)	1591 (33.2%)	1607 (33.5%)	1693 (35.3%)
Missing	728 (15.2%)	730 (15.2%)	763 (15.9%)	672 (14.0%)	647 (13.5%)
**Achieved UK physical activity recommendations via MVPA only**					
Yes	3263 (67.9%)	3339 (69.5%)	3352 (69.9%)	3459 (72.1%)	3571 (74.4%)
No	812 (16.9%)	731 (15.2%)	682 (14.2%)	666 (13.9%)	580 (12.1%)
Missing	729 (15.2%)	731 (15.2%)	764 (15.9%)	672 (14.0%)	648 (13.5%)
**Achieved UK physical activity recommendations via MVPA only**					
Yes	2023 (42.1%)	2118 (44.1%)	2135 (44.5%)	2136 (44.5%)	2245 (46.8%)
No	2053 (42.7%)	1953 (40.7%)	1900 (39.6%)	1989 (41.5%)	1907 (39.7%)
Missing	728 (15.2%)	730 (15.2%)	763 (15.9%)	672 (14.0%)	647 (13.5%)
**Non-commuting transport**					
Car/motor vehicle	3719 (77.4%)	3273 (68.2%)	2848 (59.4%)	2464 (51.4%)	1717 (35.8%)
Cycle	76 (1.6%)	99 (2.1%)	123 (2.6%)	128 (2.7%)	250 (5.2%)
Walk	616 (12.8%)	863 (18.0%)	1143 (23.8%)	1359 (28.3%)	1780 (37.1%)
Public transport	382 (8.0%)	551 (11.5%)	665 (13.9%)	834 (17.4%)	1042 (21.7%)
Missing	11 (0.2%)	15 (0.3%)	19 (0.4%)	12 (0.3%)	10 (0.2%)
**Commuting transport**					
Car/motor vehicle	3176 (66.1%)	2789 (58.1%)	2399 (50.0%)	1825 (38.0%)	1185 (24.7%)
Cycle	110 (2.3%)	171 (3.6%)	192 (4.0%)	230 (4.8%)	374 (7.8%)

Walk	596 (12.4%)	711 (14.8%)	831 (17.3%)	1037 (21.6%)	1464 (30.5%)
Public transport	922 (19.2%)	1130 (23.5%)	1376 (28.7%)	1705 (35.5%)	1776 (37.0%)

Table S15: Baseline characteristics of UK Biobank participants with commuting transport information and who commuted at least once per week (n = 23,999) by quintiles of *green walkability*; all study participants resided within Greater London administrative boundaries at study baseline (2006-2010).

	**Green walkability quintiles**				
	**Q1**	**Q2**	**Q3**	**Q4**	**Q5**
	**(N=4806)**	**(N=4798)**	**(N=4797)**	**(N=4801)**	**(N=4797)**
**Age at baseline (years)**					
Mean (SD)	53.6 (7.23)	52.6 (7.26)	52.5 (7.40)	52.5 (7.30)	52.5 (7.46)
**Sex**					
Female	2612 (54.3%)	2538 (52.9%)	2595 (54.1%)	2578 (53.7%)	2602 (54.2%)
Male	2194 (45.7%)	2260 (47.1%)	2202 (45.9%)	2223 (46.3%)	2195 (45.8%)
**Average total household income before tax (£)**					
Less than 18,000	277 (5.8%)	392 (8.2%)	399 (8.3%)	373 (7.8%)	389 (8.1%)
18,000 to 30,999	739 (15.4%)	801 (16.7%)	848 (17.7%)	794 (16.5%)	803 (16.7%)
31,000 to 51,999	1235 (25.7%)	1259 (26.2%)	1203 (25.1%)	1181 (24.6%)	1084 (22.6%)
52,000 to 100,000	1515 (31.5%)	1387 (28.9%)	1217 (25.4%)	1254 (26.1%)	1270 (26.5%)
Greater than 100,000	490 (10.2%)	457 (9.5%)	648 (13.5%)	746 (15.5%)	878 (18.3%)
Missing	550 (11.4%)	502 (10.5%)	482 (10.0%)	453 (9.4%)	373 (7.8%)
**Index of Multiple Deprivation (small-area level)**					
Low deprivation	455 (9.5%)	871 (18.2%)	1050 (21.9%)	1353 (28.2%)	2412 (50.3%)
Medium deprivation	1689 (35.1%)	2260 (47.1%)	2376 (49.5%)	2269 (47.3%)	2073 (43.2%)
High deprivation	2662 (55.4%)	1667 (34.7%)	1371 (28.6%)	1179 (24.6%)	312 (6.5%)
**IPAQ activity group**					
Low	768 (16.0%)	740 (15.4%)	697 (14.5%)	638 (13.3%)	576 (12.0%)
Moderate	1758 (36.6%)	1785 (37.2%)	1784 (37.2%)	1810 (37.7%)	1922 (40.1%)
High	1516 (31.5%)	1536 (32.0%)	1591 (33.2%)	1660 (34.6%)	1678 (35.0%)
Missing	764 (15.9%)	737 (15.4%)	725 (15.1%)	693 (14.4%)	621 (12.9%)
**Achieved UK physical activity recommendations via MVPA only**					
Yes	3276 (68.2%)	3289 (68.5%)	3368 (70.2%)	3467 (72.2%)	3584 (74.7%)
No	764 (15.9%)	772 (16.1%)	704 (14.7%)	640 (13.3%)	591 (12.3%)
Missing	766 (15.9%)	737 (15.4%)	725 (15.1%)	694 (14.5%)	622 (13.0%)
**Achieved UK physical activity recommendations via MVPA only**					
Yes	2030 (42.2%)	2051 (42.7%)	2134 (44.5%)	2174 (45.3%)	2268 (47.3%)
No	2012 (41.9%)	2010 (41.9%)	1938 (40.4%)	1934 (40.3%)	1908 (39.8%)
Missing	764 (15.9%)	737 (15.4%)	725 (15.1%)	693 (14.4%)	621 (12.9%)
**Non-commuting transport**					
Car/motor vehicle	3646 (75.9%)	3169 (66.0%)	2828 (59.0%)	2519 (52.5%)	1859 (38.8%)
Cycle	61 (1.3%)	114 (2.4%)	116 (2.4%)	149 (3.1%)	236 (4.9%)
Walk	665 (13.8%)	923 (19.2%)	1120 (23.3%)	1347 (28.1%)	1706 (35.6%)
Public transport	422 (8.8%)	568 (11.8%)	721 (15.0%)	775 (16.1%)	988 (20.6%)
Missing	12 (0.2%)	24 (0.5%)	12 (0.3%)	11 (0.2%)	8 (0.2%)
**Commuting transport**					
Car/motor vehicle	3098 (64.5%)	2717 (56.6%)	2301 (48.0%)	1918 (40.0%)	1340 (27.9%)
Cycle	109 (2.3%)	167 (3.5%)	189 (3.9%)	249 (5.2%)	363 (7.6%)
Walk	623 (13.0%)	716 (14.9%)	871 (18.2%)	1070 (22.3%)	1359 (28.3%)
Public transport	976 (20.3%)	1198 (25.0%)	1436 (29.9%)	1564 (32.6%)	1735 (36.2%)

Table S16: Baseline characteristics of UK Biobank participants with commuting transport information and who commuted at least once per week (n = 23,999) by quintiles of *ground-cover walkability*; all study participants resided within Greater London administrative boundaries at study baseline (2006-2010).

	**Ground-cover walkability quintiles**				
	**Q1**	**Q2**	**Q3**	**Q4**	**Q5**
	**(N=4806)**	**(N=4799)**	**(N=4795)**	**(N=4799)**	**(N=4800)**
**Age at baseline (years)**					
Mean (SD)	53.9 (7.21)	52.6 (7.29)	52.5 (7.36)	52.3 (7.31)	52.4 (7.41)
**Sex**					
Female	2627 (54.7%)	2528 (52.7%)	2548 (53.1%)	2588 (53.9%)	2634 (54.9%)
Male	2179 (45.3%)	2271 (47.3%)	2247 (46.9%)	2211 (46.1%)	2166 (45.1%)

**Average total household income before tax (£)**					
Less than 18,000	232 (4.8%)	358 (7.5%)	425 (8.9%)	428 (8.9%)	387 (8.1%)
18,000 to 30,999	651 (13.5%)	770 (16.0%)	880 (18.4%)	873 (18.2%)	811 (16.9%)
31,000 to 51,999	1167 (24.3%)	1239 (25.8%)	1255 (26.2%)	1196 (24.9%)	1105 (23.0%)
52,000 to 100,000	1617 (33.6%)	1390 (29.0%)	1187 (24.8%)	1188 (24.8%)	1261 (26.3%)
Greater than 100,000	652 (13.6%)	582 (12.1%)	532 (11.1%)	628 (13.1%)	825 (17.2%)
Missing	487 (10.1%)	460 (9.6%)	516 (10.8%)	486 (10.1%)	411 (8.6%)
**Index of Multiple Deprivation (small-area level)**					
Low deprivation	319 (6.6%)	786 (16.4%)	1114 (23.2%)	1576 (32.8%)	2346 (48.9%)
Medium deprivation	1546 (32.2%)	2200 (45.8%)	2456 (51.2%)	2445 (50.9%)	2020 (42.1%)
High deprivation	2941 (61.2%)	1813 (37.8%)	1225 (25.5%)	778 (16.2%)	434 (9.0%)
**IPAQ activity group**					
Low	780 (16.2%)	714 (14.9%)	699 (14.6%)	672 (14.0%)	554 (11.5%)
Moderate	1834 (38.2%)	1782 (37.1%)	1773 (37.0%)	1767 (36.8%)	1903 (39.6%)
High	1511 (31.4%)	1583 (33.0%)	1568 (32.7%)	1620 (33.8%)	1699 (35.4%)
Missing	681 (14.2%)	720 (15.0%)	755 (15.7%)	740 (15.4%)	644 (13.4%)
**Achieved UK physical activity recommendations via MVPA only**					
Yes	3346 (69.6%)	3337 (69.5%)	3327 (69.4%)	3393 (70.7%)	3581 (74.6%)
No	778 (16.2%)	741 (15.4%)	712 (14.8%)	665 (13.9%)	575 (12.0%)
Missing	682 (14.2%)	721 (15.0%)	756 (15.8%)	741 (15.4%)	644 (13.4%)
**Achieved UK physical activity recommendations via MVPA only**					
Yes	2045 (42.6%)	2110 (44.0%)	2081 (43.4%)	2145 (44.7%)	2276 (47.4%)
No	2080 (43.3%)	1969 (41.0%)	1959 (40.9%)	1914 (39.9%)	1880 (39.2%)
Missing	681 (14.2%)	720 (15.0%)	755 (15.7%)	740 (15.4%)	644 (13.4%)
**Non-commuting transport**					
Car/motor vehicle	3604 (75.0%)	3101 (64.6%)	2851 (59.5%)	2526 (52.6%)	1939 (40.4%)
Cycle	61 (1.3%)	130 (2.7%)	121 (2.5%)	136 (2.8%)	228 (4.8%)
Walk	694 (14.4%)	994 (20.7%)	1098 (22.9%)	1294 (27.0%)	1681 (35.0%)
Public transport	437 (9.1%)	554 (11.5%)	710 (14.8%)	829 (17.3%)	944 (19.7%)
Missing	10 (0.2%)	20 (0.4%)	15 (0.3%)	14 (0.3%)	8 (0.2%)
**Commuting transport**					
Car/motor vehicle	2987 (62.2%)	2617 (54.5%)	2378 (49.6%)	1980 (41.3%)	1412 (29.4%)
Cycle	116 (2.4%)	197 (4.1%)	180 (3.8%)	235 (4.9%)	349 (7.3%)
Walk	670 (13.9%)	770 (16.0%)	852 (17.8%)	1001 (20.9%)	1346 (28.0%)
Public transport	1033 (21.5%)	1215 (25.3%)	1385 (28.9%)	1583 (33.0%)	1693 (35.3%)

Table S17: Baseline characteristics of UK Biobank participants with commuting transport information and who commuted at least once per week (n = 23,999) by quintiles of *tree-cover walkability*; all study participants resided within Greater London administrative boundaries at study baseline (2006-2010).

	**Ground-cover walkability quintiles**				
	**Q1**	**Q2**	**Q3**	**Q4**	**Q5**
	**(N=4806)**	**(N=4799)**	**(N=4795)**	**(N=4799)**	**(N=4800)**
**Age at baseline (years)**					
Mean (SD)	53.9 (7.21)	52.6 (7.29)	52.5 (7.36)	52.3 (7.31)	52.4 (7.41)
**Sex**					
Female	2627 (54.7%)	2528 (52.7%)	2548 (53.1%)	2588 (53.9%)	2634 (54.9%)
Male	2179 (45.3%)	2271 (47.3%)	2247 (46.9%)	2211 (46.1%)	2166 (45.1%)
**Average total household income before tax (£)**					
Less than 18,000	232 (4.8%)	358 (7.5%)	425 (8.9%)	428 (8.9%)	387 (8.1%)
18,000 to 30,999	651 (13.5%)	770 (16.0%)	880 (18.4%)	873 (18.2%)	811 (16.9%)
31,000 to 51,999	1167 (24.3%)	1239 (25.8%)	1255 (26.2%)	1196 (24.9%)	1105 (23.0%)
52,000 to 100,000	1617 (33.6%)	1390 (29.0%)	1187 (24.8%)	1188 (24.8%)	1261 (26.3%)
Greater than 100,000	652 (13.6%)	582 (12.1%)	532 (11.1%)	628 (13.1%)	825 (17.2%)
Missing	487 (10.1%)	460 (9.6%)	516 (10.8%)	486 (10.1%)	411 (8.6%)
**Index of Multiple Deprivation (small-area level)**					
Low deprivation	319 (6.6%)	786 (16.4%)	1114 (23.2%)	1576 (32.8%)	2346 (48.9%)
Medium deprivation	1546 (32.2%)	2200 (45.8%)	2456 (51.2%)	2445 (50.9%)	2020 (42.1%)
High deprivation	2941 (61.2%)	1813 (37.8%)	1225 (25.5%)	778 (16.2%)	434 (9.0%)
**IPAQ activity group**					
Low	780 (16.2%)	714 (14.9%)	699 (14.6%)	672 (14.0%)	554 (11.5%)
Moderate	1834 (38.2%)	1782 (37.1%)	1773 (37.0%)	1767 (36.8%)	1903 (39.6%)

High	1511 (31.4%)	1583 (33.0%)	1568 (32.7%)	1620 (33.8%)	1699 (35.4%)
Missing	681 (14.2%)	720 (15.0%)	755 (15.7%)	740 (15.4%)	644 (13.4%)
**Achieved UK physical activity recommendations via MVPA only**					
Yes	3346 (69.6%)	3337 (69.5%)	3327 (69.4%)	3393 (70.7%)	3581 (74.6%)
No	778 (16.2%)	741 (15.4%)	712 (14.8%)	665 (13.9%)	575 (12.0%)
Missing	682 (14.2%)	721 (15.0%)	756 (15.8%)	741 (15.4%)	644 (13.4%)
**Achieved UK physical activity recommendations via MVPA only**					
Yes	2045 (42.6%)	2110 (44.0%)	2081 (43.4%)	2145 (44.7%)	2276 (47.4%)
No	2080 (43.3%)	1969 (41.0%)	1959 (40.9%)	1914 (39.9%)	1880 (39.2%)
Missing	681 (14.2%)	720 (15.0%)	755 (15.7%)	740 (15.4%)	644 (13.4%)
**Non-commuting transport**					
Car/motor vehicle	3604 (75.0%)	3101 (64.6%)	2851 (59.5%)	2526 (52.6%)	1939 (40.4%)
Cycle	61 (1.3%)	130 (2.7%)	121 (2.5%)	136 (2.8%)	228 (4.8%)
Walk	694 (14.4%)	994 (20.7%)	1098 (22.9%)	1294 (27.0%)	1681 (35.0%)
Public transport	437 (9.1%)	554 (11.5%)	710 (14.8%)	829 (17.3%)	944 (19.7%)
Missing	10 (0.2%)	20 (0.4%)	15 (0.3%)	14 (0.3%)	8 (0.2%)
**Commuting transport**					
Car/motor vehicle	2987 (62.2%)	2617 (54.5%)	2378 (49.6%)	1980 (41.3%)	1412 (29.4%)
Cycle	116 (2.4%)	197 (4.1%)	180 (3.8%)	235 (4.9%)	349 (7.3%)
Walk	670 (13.9%)	770 (16.0%)	852 (17.8%)	1001 (20.9%)	1346 (28.0%)
Public transport	1033 (21.5%)	1215 (25.3%)	1385 (28.9%)	1583 (33.0%)	1693 (35.3%)
